# Draft Genome Sequence of Serratia marcescens SSA1, Isolated from Dioxin-Polluted Soil

**DOI:** 10.1128/mra.00236-22

**Published:** 2022-06-27

**Authors:** Salametu Saibu, Nwanneka M. Akinyemi, Ayodeji A. Odunsi, Imade Y. Nsa, Ganiyu O. Oyetibo, Sunday A. Adebusoye

**Affiliations:** a Department of Microbiology, Faculty of Science, University of Lagos, Lagos, Nigeria; University of Southern California

## Abstract

Serratia marcescens SSA1 was isolated from a dump site with a history of incineration. Its DNA of 5.05 Mbp has a GC content of 59.65%, with 77 tRNA genes and 3 rRNA genes. Its 4,909 putative PATRIC protein-coding genes include genes responsible for the degradation of dioxins and other xenobiotics and total consumption of benzoate.

## ANNOUNCEMENT

The rod-shaped Gram-negative enteric bacterium Serratia marcescens has been reported to metabolize different polycyclic aromatic hydrocarbons (PAHs), heavy metals, and antibiotics (1). S. marcescens SSA1 was discovered in a screen for dioxin-degrading bacteria in dibenzofuran-rich soil obtained from an industrial waste dump site that had been repeatedly burned (2).

The strain was isolated using an enrichment technique in the presence of dioxin congeners, and it could break down dibenzofuran, dichlorodibenzofuran, and dichlorodibenzo-*p*-dioxin (2). Its identity was confirmed by 16S rRNA gene sequencing (GenBank accession number MH714861) and biochemical characterization (2). It was selected for whole-genome sequencing to identify the genes linked to dioxin degradation and to determine whether other genes associated with catabolic pathways for xenobiotic compounds were present.

The genomic DNA of S. marcescens SSA1 was extracted from a fresh axenic LB culture grown overnight at 28°C using the Quick-DNA fungal/bacterial miniprep kit (Zymo Research, Irvine, CA, USA), and the DNA was quantified using a QuantiFluor double-stranded DNA (dsDNA) system (catalogue number E2670; Promega) on a Qubit 3.0 fluorometer. The library preparation and 2 × 150-bp paired-end sequencing were done using the Nextera XT Index kit v2 (FC-131-2001 to -2004) and a NextSeq 500 system (Illumina, San Diego, CA, USA) at Quadram Institute Bioscience (Norwich, UK).

The Trim Galore tool v0.6.5 (3) was employed for preassembly trimming, and the quality of the reads was assessed using QUAST v5.0.2 (4, 5). The number of reads generated was 797,318, with an average length of 149 bp. Genome assembly was done using Unicycler v0.4.8 (6), and postassembly polishing was performed using Pilon v1.23 (7). The assembled contigs were annotated using RASTtk v1.073 (8) on the Pathosystems Resource Integration Center (PATRIC) server v3.6.9 (9). All tools were run on PATRICbrc with default parameters.

The genome sequence of S. marcescens SSA1 comprised a total of 5,051,852 bp, with a sequencing depth of 45.538×, forming 50 contigs, with a GC content of 59.65%. The longest contig was 444,247 bp, and it had an *N*_50_ value of 218,827 bp. After annotation using RASTtk, there were 4,121 PATRIC protein-coding sequences and 788 hypothetical proteins, 77 tRNA genes, and 3 rRNA genes.

The S. marcescens SSA1 genome possesses genes with putative functions in the degradation of dioxin, anthracene, naphthalene, phenanthrene, toluene, trinitrotoluene, styrene, and xylene. Notably, it has the complete suite of genes for benzoate degradation via hydroxylation (pathway identification number 00362), allophanate hydrolase (EC 3.5.1.54) (PLF_613_00003067), which catalyzes mineralization in the atrazine pathway, α-keto acid decarboxylase (EC 4.1.1.−), which also transforms bis(*p*-chlorophenyl)acetic acid (DDA) to bis(*p*-chlorophenyl)methane (DDM) in 1,1,1-trichloro-2,2-bis(*p*-chlorophenyl)ethane (DDT) degradation, and cytochrome P450 enzymes (EC 2.5.1.18 [PLF_613_00002361] and EC 1.1.1.1 [PLF_613_00002723]) responsible for biotransformation of xenobiotics.

In general, the genome sequence data indicate that the catabolic capabilities of S. marcescens SSA1 are spread across dioxins, pesticides, and PAHs, thus reinforcing the metabolic versatility of this strain and its potential for the remediation of soils contaminated with an array of xenobiotic compounds.

### Data availability.

The draft genome annotation of Serratia marcescens SSA1 was run on PATRICbrc under the accession number 615.2149. The draft genome sequence was deposited in GenBank under the accession number JALPZJ000000000; project data are available under BioProject accession number PRJNA782969, with BioSample accession number SAMN23408247 and SRA accession number SRR17017416.
